# Rumen microbiota of indigenous and introduced ruminants and their adaptation to the Qinghai–Tibetan plateau

**DOI:** 10.3389/fmicb.2022.1027138

**Published:** 2022-10-10

**Authors:** Bin Li, Gaobin Jia, Dongxu Wen, Xiuxin Zhao, Junxing Zhang, Qing Xu, Xialing Zhao, Nan Jiang, Zhenjiang Liu, Yachun Wang

**Affiliations:** ^1^Institute of Animal Husbandry and Veterinary, Tibet Academy of Agricultural and Animal Husbandry Sciences, Lhasa, China; ^2^Agricultural College, Ningxia University, Yinchuan, China; ^3^Colleges of Life Science and Technology, Dalian University, Dalian Economic Technological Development Zone, Dalian, China; ^4^Institute of Life Sciences and Bio-Engineering, Beijing Jiaotong University, Beijing, China; ^5^National Engineering Laboratory for AIDS Vaccine, School of Life Sciences, Jilin University, Changchun, China; ^6^Laboratory of Animal Genetics, Breeding and Reproduction, Ministry of Agriculture of China, National Engineering Laboratory of Animal Breeding, College of Animal Science and Technology, China Agricultural University, Beijing, China

**Keywords:** plateau, adaptation, ruminants, microbiota, interaction

## Abstract

The grassland in the Qinghai–Tibetan plateau provide habitat for many indigenous and introduced ruminants which perform important ecological functions that impact the whole Qinghai–Tibetan plateau ecosystem. These indigenous Tibetan ruminants have evolved several adaptive traits to withstand the severe environmental conditions, especially cold, low oxygen partial pressure, high altitude, strong UV radiation, and poor forage availability on the alpine rangelands. Despite the challenges to husbandry associated with the need for enhanced adaptation, several domesticated ruminants have also been successfully introduced to the alpine pasture regions to survive in the harsh environment. For ruminants, these challenging conditions affect not only the host, but also their commensal microbiota, especially the diversity and composition of the rumen microbiota; multiple studies have described tripartite interactions among host-environment-rumen microbiota. Thus, there are significant benefits to understanding the role of rumen microbiota in the indigenous and introduced ruminants of the Qinghai–Tibetan plateau, which has co-evolved with the host to ensure the availability of specific metabolic functions required for host survival, health, growth, and development. In this report, we systemically reviewed the dynamics of rumen microbiota in both indigenous and introduced ruminants (including gut microbiota of wild ruminants) as well as their structure, functions, and interactions with changing environmental conditions, especially low food availability, that enable survival at high altitudes. We summarized that three predominant driving factors including increased VFA production, enhanced fiber degradation, and lower methane production as indicators of higher efficiency energy harvest and nutrient utilization by microbiota that can sustain the host during nutrient deficit. These cumulative studies suggested alteration of rumen microbiota structure and functional taxa with genes that encode cellulolytic enzymes to potentially enhance nutrient and energy harvesting in response to low quality and quantity forage and cold environment. Future progress toward understanding ruminant adaptation to high altitudes will require the integration of phenotypic data with multi-omics analyses to identify host-microbiota co-evolutionary adaptations enabling survival on the Qinghai–Tibetan plateau.

## Introduction

Known as “the roof of the world” and the “Water Tower of Asia,” the Qinghai–Tibetan Plateau (QTP) is the highest and largest plateau in the world, averaging 4,500 m above sea level and covering more than 2.5 million km^2^. Water supply from the QTP thus affects roughly 40% of the global population that reside in the surrounding lowlands (Yu et al., 2020; Jing et al., 2022). A large proportion of QTP residents live at altitudes over 3,500 m, and more than 90% of this population are engaged in farming and herding (Wu, 2001). The domesticated ruminants that primarily graze on pasture provide the local herders with milk, meat, fuel (yak dung), and wool (Li et al., 2018). Together with wild ruminants, these livestock perform important ecological functions that impact the whole QTP ecosystem.

The grassland ecosystems above 3,500 m cover about 60% of the QTP and provide an essential habitat for many ruminant species that utilize pasture for grazing throughout the year (Long et al., 1999). In addition to wild ruminants such as Tibetan antelope (*Pantholops hodgsoni*), wild yak (*Bos mutus*), and Tibetan gazelle (*Procapra picticaudata*), large numbers of domesticated indigenous ruminants including yak (*Bos grunniens*), Tibetan sheep (*Ovis aries*), and goats (*Capra hircus*) graze on the native grasslands of the plateau. These indigenous Tibetan ruminants have evolved several adaptive traits to withstand the severe environmental conditions, especially cold, with night-time temperatures averaging around −20°C and routinely dropping below −30°C in winter (Wang et al., 2004). In addition, low oxygen partial pressure, high altitude, strong UV radiation, Low precipitation, and poor forage availability on the alpine rangelands all present physiological challenges for its fauna (Long et al., 1999; Yao et al., 2012; Liu et al., 2016; Friedrich and Wiener, 2020; Jing et al., 2022). Despite the challenges to husbandry associated with the need for enhanced adaptation, several domesticated ruminants have also been successfully introduced to the alpine pasture regions for meat and dairy production, such as hybrid sheep, goats, and cattle (Huang et al., 2016; Zhang et al., 2022).

In order to adapt to the high altitude, both humans and animals have evolved physiologically and morphologically to survive in the harsh environment. Mammals exhibit several physiological responses during exposure to high altitudes, such as increased oxygen transport in the blood mediated by enhanced hemoglobin function, which enables a higher performance capacity (i.e., VO_2_ max) to sustain life in low oxygen conditions (Storz, 2021). The genetic background underlying adaptation to high altitudes has been well-studied in both humans and animals, including ruminants (Beall, 2014; Friedrich and Wiener, 2020; Sharma et al., 2022). For ruminants, these challenging conditions affect not only the host, but also their commensal microbiota, especially the diversity and composition of the rumen microbiota; multiple studies have described tripartite interactions among host-environment-rumen microbiota.

Ruminants graze on a wide variety of grasses, sedges, and some shrubs on the QTP, which are collectively considered forage species. The rumen functions as a large anaerobic fermentation tank that harbors diverse, symbiotic bacteria, fungi, archaea, and protozoa which together effectively degrade complex plant fibers and polysaccharides in straw, silage, hay, and grass, in turn producing volatile fatty acids, vitamins, and microbial proteins that can be utilized by ruminants to meet their nutritional requirements. Rumen microbiota are distinct from other commensal communities in their high population density, diversity, and relatively complex interaction networks that involve competition, mutualism, predation, parasitism, and amensalism (Faust and Raes, 2012; McCann et al., 2014). The fermentative metabolic functions by rumen microbiota led to the mineralization of large saccharide chains in feed into small diffusible components. Volatile fatty acids (energy source), carbon dioxide (electron acceptor), and hydrogen (electron donor) all accumulate in the rumen through the fermentation of feed (Morgavi et al., 2010). Thus, there are significant benefits to understanding the role of rumen microbiota in ruminant metabolism, since rumen dynamics are almost exclusively responsible for supplying the host animal with nutrients (Krehbiel, 2014).

**FIGURE 1:**
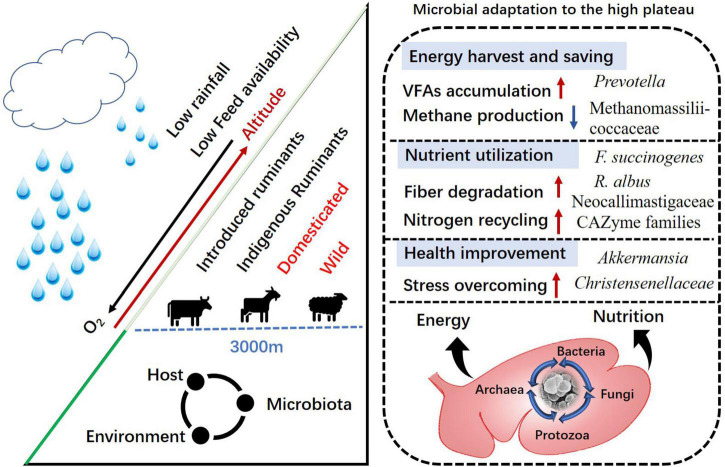
Rumen microbial adaptation to the Qinghai–Tibetan plateau among indigenous and introduced ruminants.

The rumen microbial community has typically high diversity, which has co-evolved with the host for millions of years to ensure the availability of specific metabolic functions required for host survival, health, growth, and development (Popkes and Valenzano, 2020). Along with bacteria, there are fungi, methanogenic archaea, and protozoa that also contribute to environmental adaptation by indigenous ruminants of the QTP. The vegetation period on the QTP is around 100–150 days, while the dormant period lasts for roughly 7 months, with characteristically harsh weather conditions (Xue et al., 2005). Grasses sprout in early May, with the highest biomass occurring between late August and early September (Wang et al., 2013). These seasonal changes in forage biomass hugely impacts the physiological performance of ruminants, and thus they have acquired a complex system of physiological, morphological, and microbial adaptations to mitigate the environmental impacts (Friedrich and Wiener, 2020; Liu et al., 2021). To help further our understanding of rumen microbiota in the indigenous and introduced ruminants of the QTP, in this review, we summarize their structure and interactions with changing environmental conditions, especially low food availability, that enable survival at high altitudes (Figure 1).

## Overview of rumen/gut microbiota in wild and domesticated indigenous ruminants of the Qinghai–Tibetan plateau

### Wild ruminants (Tibetan antelope, Tibetan gazelle, and wild yak)

There are several wild ruminants indigenous to the QTP, such as Tibetan antelope (*Pantholops hodgsoni*), wild yak (*Bos mutus*), and Tibetan gazelle (*Procapra picticaudata*) (Zhang et al., 2002; Leslie, 2010). In summer, these ruminants spend the majority of their time in alpine deserts at 5,000 MASL. During winter, as temperatures and food availability decrease, they migrate to lower elevations (Jing et al., 2022). These rare ruminants appear on the International Union for Conversation of Nature (IUCN) 2012 Red List of threatened species, and research has been conducted to promote their conservation (Dong et al., 2015). Due to their rarity, gut microbiota studies in these animals rely on fecal biological samples and therefore can only partially reflect the rumen microbial ecosystem, although gut microbiota can be informative of digestive conditions in the rumen and play key roles in ruminant metabolism.

Low temperatures and hypoxia on the QTP are extreme environmental stimuli for wild Caprinae species such as Tibetan antelope and Tibetan gazelle that are widely distributed but unique to the plateau. Tibetan antelope has evolved an increase in blood-O_2_ affinity that is an irreversible adaptation to chronic hypoxia (Signore and Storz, 2020). Moreover, these animals survive only on natural vegetation through the barren season of the grassland with scarce forage availability, dependent on the efficiency of their commensal microbiota for the digestion of nutrients from low quality feeds (Sahu and Kamra, 2002). These wild ruminants are thus widely believed to display a high tolerance for low nutrient forage through their unique microbial communities. For instance, Bai et al. (2018) found a large number of uncultured taxa in metagenomics sequence data of fecal microbiota of Tibetan Antelope. In particular, this study detected a prevalence of uncultured Ruminococcaceae, Christensenellaceae, and Lachnospiraceae, as well as uncultured *Bacteroides* and *Akkermansia* which together form the core taxa most likely responsible for high-efficiency degradation of recalcitrant plant matter. Moreover, these taxa have been recently identified as biomarkers of a healthy mucus layer in the animal gut that may provide a competitive advantage during nutrient deprivation and are associated with longevity in humans (Belzer and de Vos, 2012; Biagi et al., 2016).

Shi et al. (2021) examined changes in the gut microbiota of Tibetan Antelope during the peri-parturition period and reported Firmicutes and Bacteroidetes as predominant phyla. In addition, these taxa shift during the transition from late pregnancy to the postpartum period, and microbial diversity is correlated with glucocorticoid and triiodothyronine levels to further accommodate energy demands and immune system modulation. Shang et al. (2022) characterized the diversity and composition of gut fungi in the Tibetan antelope and Tibetan gazelle. This study reported that fungal diversity (Shannon index) was significantly lower in the Tibetan antelope than in the Tibetan gazelle (4.64 vs. 2.67) and further suggested host variations for microbial alpha diversity, both gut fungi were dominated by the Ascomycota and Basidiomycota phyla, which, respectively, comprised 74.94 and 21.56% of the fungal population in Tibetan gazelle and 66.24 and 32.68% of the fungal population in Tibetan antelope. These findings suggested that metabolic interactions between gut fungi and the host help possibly cope with low forage availability, largely through a high potential for cellulose degradation. Thus, these microorganisms enhance the digestibility of dry, low nutrient forage matter in Tibetan gazelle and Tibetan antelope (Cao et al., 2007).

One study of wild yaks identified an increase in microbial diversity and enrichment for Firmicutes compared to that in domestic yak, as well as increased abundance of microbial pathways involved in digestion and enrichment for rapidly evolved genes in energy and carbohydrate metabolism pathways, most notably cellulase and endohemicellulase glycoside hydrolases (Fu et al., 2021). These findings aligned well with a previous study that highlighted the contribution of Firmicutes to efficient energy harvesting (Nicholson et al., 2012), which together strongly suggested that fecal microbiota of wild yak most likely co-evolved with their host to mediate efficient cellulose utilization. However, there are remarkably few microbiota studies in wild yak and therefore little information or evidence to infer their microbiota-host interactions. In addition, the conclusions that can be drawn regarding their microbial ecosystems based on fecal samples are limited in the absence of direct supporting data from rumen microbiota.

### Domesticated ruminants (yak, Tibetan sheep, and indigenous cattle)

Through long-term natural selection and artificial breeding, indigenous QTP ruminant livestock (yak, Tibetan sheep, and indigenous cattle) have developed strong ecological adaptations to the high plateau, while also playing key roles in the ecological stability of the QTP. Sociologically, these animals also drive the economy, provide essential materials for human life, and are considered a prized feature of the QTP cultural heritage.

**FIGURE 2:**
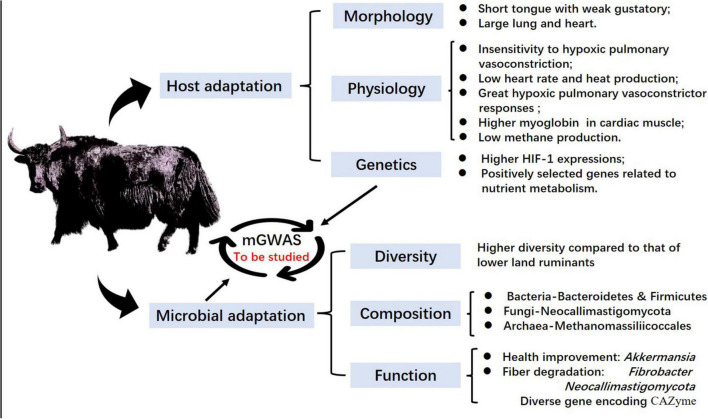
Yak adaptation to the Qinghai–Tibetan plateau in the view of both host and rumen microbiota.

Yaks are large ruminants in the Qinghai–Tibetan plateau that can effectively utilize alpine meadow and help improve the structure and function of alpine grasslands by maintaining ecosystem stability. Both Qiu et al. (2015) and Ma et al. (2018) reported that wild yaks were initially domesticated between 5,000 and 7,300 years ago, and gradually transitioned into modern domestic yaks on the QTP. This domestication event likely provided a foundation for the large-scale expansion and permanent settlement of prehistoric humans on the QTP roughly 3,600 years ago (Chen et al., 2015). Therefore, through thousands of years of husbandry, yaks have developed marked ecological adaptations and unique biological traits that are intimately linked to their economic and ecological importance, as well as their role in cultural identity (Long et al., 1999; Zhang et al., 2016; Zhou et al., 2017; Joshi et al., 2020). At present, approximately 16 million yaks are found on the QTP, accounting for 90% of the total global population (Wang L. Z. et al., 2017; Wang H. et al., 2019). Qiu et al. (2012, 2015) suggested that yak evolved to tolerate the harsh environment over millions of years through enrichment of protein and gene families related to hypoxia and energy metabolism. In addition, their rumen microbiota shows unique adaptations for colonization during yak development, and shifts in community composition and function in response to the harsh environment of the plateau (Figure 2).

Recent studies on host colonization by yak rumen microflora found that the establishment of these rumen microflora in calves is most affected by the maternal rumen microbiota and protozoa, with core taxa including *Christensenellaceae R-7 group*, *Prevotella* 1, *Ruminococcaceae ucg-014*, Lachnospiraceae, and *Trichostomatia* (Guo et al., 2020b). *Prevotella* has been associated with propionic acid production and this genus is known to play a pivotal role in the degradation and utilization of non-cellulosic plant polysaccharides, proteins, starches, and xylans (Strobel, 1992; Accetto and Avguštin, 2019). In adult yaks, Bacteroidetes and Firmicutes are the predominant bacterial phyla, although their proportions vary in yak in diet-dependent manner (Liu C. et al., 2019). At the genus level; *Fibrobacter*, *Lachnospira*, and *Pseudobutyrivibrio* are more abundant in yak rumen than in rumen of cattle, sheep, or Tibetan sheep (Huang et al., 2021). In contrast with rumen of dairy cows, which is enriched with phylum Ascomycota (Kumar et al., 2015), the fungal phylum Neocallimastigomycota is most prevalent in rumen of yaks and Tibetan sheep, potentially due to their role in enhanced degradation of plant material (Gruninger et al., 2014; Guo et al., 2020a). These findings illustrate the high potential for utilization of recalcitrant plant substrates by rumen microbiota of indigenous QTP ruminants. In addition, several previously undocumented fungi and archaea were found to be abundant in yak rumen (Huang et al., 2012, 2016; Guo et al., 2020b), suggesting that many as-of-yet unknown functional microbes might contribute to the tripartite interaction network among the host, environment and rumen microbiota of indigenous QTP ruminants.

Rumen microbiota play important roles in nutrient utilization, metabolism, immune function, animal health, and even host development (Sonnenburg and Bäckhed, 2016; Islam et al., 2019). Numerous studies have shown that yak rumen microbes can help to overcome stress, save energy, improve nitrogen utilization, and increase fiber degradation, thereby improving nutrient intake from forage sources (Zhang et al., 2016; Zhou et al., 2017, 2018b; Shi et al., 2020). In addition, Ding et al. (2010) reported that methane (CH_4_) emissions, which causes energy loss as a byproduct of rumen fermentation by methanogen, from grazing yaks were significantly lower than those of European and American beef cattle breeds. Similar to evidence reported by Huang et al. (2012, 2016) that yak rumen contains a unique methanogen structure compared with that of other QTP ruminants, Wang et al. (2020) deduced that differences in methanogen composition is a likely contributing factor in the lower CH_4_ production in yaks than in cattle. Both of these studies suggest that yaks have a “low carbon mode”/“energy saving mode” for persistence on the plateau that probably associated with methanogen functions. To further validate the microbial interactions in nutrient utilization, Wei Y. Q. et al., 2016; Wei et al., 2017) showed that symbiotic fungi and methanogens confer a high capacity for fiber degradation in yak rumen. These studies suggested that methanogens and methanogen interactions with other microbes could be potentially exploited or modulated to mitigate enteric CH_4_ emissions from ruminants on the QTP.

Seasonal changes also significantly influence feed and forage availability in the QTP; thus, seasonal dynamics in rumen microbiota feature shifts in specific taxa in response to extreme environmental cues. Multiple studies of seasonal variations in rumen of grazing yaks found that fiber-degrading bacteria such as *Fibrobacter succinogenes* and *Ruminococcus albus* were enriched in the cold season, potentially facilitating coping with nutritional stress (Guo et al., 2021; Huang et al., 2021). Guo et al. (2020b) also found that *Akkermansia* and *wchb1-41*, which are involved in arginine and fatty acid synthesis, are enriched in the cold season, thus enabling yak adaptation to low carbon and nitrogen intake and low feed availability. As the primary end product of carbohydrate fermentation in rumen, VFAs are closely related to rumen microbiota composition and diversity, potentially providing 80% of host energy demands (Bergman, 1990; Poudel et al., 2019), 85% of these VFAs can be transported and absorbed directly through rumen epithelium in different forms (Müller et al., 2002; Graham et al., 2007). Zhang et al. (2016) reported that specific microbial genes that are enriched in yak and Tibetan sheep rumen could be correlated with high energy-yielding pathways for VFA production, suggesting that both rumen microbiota and hosts have evolved to harvest energy more efficiently from forage, and provides important insights into convergent adaptation of indigenous ruminants to high altitude. From the perspective of energy metabolism, yak rumen microbes may have the ability to efficiently use limited nitrogen resources during periods of nutritional stress, which aligns well with reportedly high nitrogen utilization efficiency through endogenous nitrogen recycling detected through analysis of urea kinetics (Zhou et al., 2017, 2018b). These collective studies thus show that yak rumen microbiota respond to cold conditions, insufficient forage, and high fiber content feed. Future work will comprehensively and systematically characterize the effects of spatial distribution patterns in yak rumen microorganisms and their role in nitrogen utilization efficiency, which can lay a theoretical and practical foundation for understanding yak adaptations to the plateau.

Similar to yak, Tibetan sheep are also indigenous ruminants of the QTP which display unique biological characteristics and are well-adapted to tolerate the cold, low nutrition highland conditions (Zhou et al., 2018a,^2019^; Jing et al., 2019). These sheep mainly live in alpine grasslands at 3,000–5,000 MASL. At present, there are about 50 million Tibetan sheep on the Qinghai–Tibet Plateau, roughly three times greater than the yak population (Xin et al., 2011). Wei C. et al. (2016) detected selection events spanning genes involved in angiogenesis, energy production and erythropoiesis, as well as several candidate genes associated with high altitude hypoxia. During severe nutrient deficits in the cold season, Tibetan sheep display strong physiological, ecological and nutritional adaptability, and continually provide herders with high-quality meat, leather, wool, and other animal products (Suo et al., 2020).

Rumen microbiota have also been shown to participate in the adaptability of Tibetan sheep hosts. The rumen of Tibetan lambs that weaned early remain incompletely developed. Diet structure at this stage also strongly impacts rumen development. Metagenomic 16s rRNA gene sequencing of rumen bacteria from 27 Tibetan lambs at different developmental stages revealed a significant increase in Bacteroidetes and a decrease in Proteobacteria during the first year of the growth, potentially driven by both host development and dietary changes, further illustrating how environmental dependence on diet is important for colonization by rumen microbes in Tibetan sheep (Wang L. et al., 2019). Therefore, the high-efficiency feed degradation in Tibetan sheep may be related to its specialized rumen microflora, while ruminant development affects the structure and function of rumen microbiota. Rumen bacteria in adult sheep are dominated by Bacteroidetes and Firmicutes, as in other species, with *Prevotella_1* and *Rikenellaceae_RC9_gut_group* representing the dominant genera. *Prevotella* has been associated with VFA production and plays a pivotal role in nutrient utilization in Tibetan sheep (Liu H. et al., 2019). Apart from host effects, dietary fluctuations on the high plateau are a main driver shaping rumen microbiota structure. Cui et al. (2019) reported that the rumen of Tibetan sheep fed with oat hay had higher proportions of Proteobacteria than the rumen of grazing in natural pastures. Metagenomics analysis of Tibetan sheep rumen microbiota from different stages of pasture phenology revealed enrichment with *Bacteroidetes*, *Prevotella*, *Succiniclasticum*, and *Treponema* in growing season, characterized by high nutritional quality forage, which were associated with high concentrations of NH_3_ nitrogen and elevated VFA production (Liu H. et al., 2020). Similar to results in yak (Huang et al., 2021), *Verrucomicrobia*, which plays an important role in polysaccharide and cellobiose decomposition, were increased in Tibetan sheep in the barren season (Godoy-Vitorino et al., 2012; Gharechahi et al., 2015; Liu H. et al., 2020). Furthermore, studies examining the effects of feeding patterns on rumen prokaryotic communities in grazing or mixed ration-fed Tibetan sheep found that *Methanimicrococcus* was only present in the grazing sheep, while the abundance of *Methanosphaera*, *unclassified BS11*, *BF311*, *CF231* groups, and *Shuttleworthia* were also significantly higher in the grazing groups than in the total mixed ration groups (Xue et al., 2017). These results confirmed that feeding patterns can modulate rumen prokaryotic community composition.

As with other indigenous QTP ruminants, fungal communities are dominated by Ascomycota (69.56%) and Basidiomycota (25.16%) in the fecal samples of Tibetan sheep, with Cordycipitaceae (11.78%) and Ustilaginaceae (7.44%) identified as the most prevalent families (Shang et al., 2022). In rumen samples of grazing Tibetan sheep, Neocallimastigaceae is the most abundant family, accounting for 97.44% of the total fungi, and likely plays a similar role in fiber degradation to that proposed in other QTP ruminants (Guo et al., 2020a). Liu X. et al. (2020) reported higher concentrations of VFAs and higher microbial richness in Tibetan sheep in the cold season than in the warm season, as well as correlation among VFAs, specific microbial taxa, and enriched host genes related to nutrient absorption and rumen epithelial barrier function. These findings suggested that rumen fermentation parameters, rumen microbes, and host gene expression of jointly contribute to nutrient absorption and rumen epithelial barrier function of plateau ruminants in harsh environments.

Other than these cellulolytic bacteria in the rumen, the diversity of the prevalent rumen microorganisms and putative carbohydrate-active enzymes for utilization of lignocellulosic biomass are also important in host adaptation of high plateau (Bohra et al., 2019). Gong et al. (2020) suggested complex gene repertoire composed of diverse carbohydrate-degrading enzymes for yak gut microbiota which provide multiple catalytic abilities of various carbohydrate substrates deconstruction. Zhao et al. (2022) also reported a larger gene pool encoding rich in carbohydrate-active enzymes in the yak rumen microbiota by metagenomic analysis. For the rumen microbiota of Tibetan sheep, enrichment with genes (GHs and CBMs) related to cellulolytic enzymes, metabolic pathways, fatty acid biosynthesis, and antibiotics biosynthesis could also help hosts overcome harsh environment and low forage availability. In addition, energy-metabolism-related genes in conjunction with adaptive evolution could also help these indigenous ruminants living on the QTP (Qiu et al., 2012; Ge et al., 2013). These studies provide genetic profiles for enzymes and microbial candidate involved in the degradation of complex plant polysaccharides and thus give insight of fiber degradation function of rumen microbiota and their contribution to the microbial adaptation of high plateau in a view of enhancing food availability.

In addition to yaks and Tibetan sheep, indigenous Chinese cattle breeds in the highland also exhibit diverse environmental adaptations. One study reported that Tibetan, Apeijiaza, and Shigatse humped cattle displayed high tolerance to low pressure and low oxygen, and possibly linked to enrichment with CNVs in NOXA1, RUVBL1, and SLC4A3, which may play important roles in adaptation to high altitude environments (Zhang et al., 2019). Another bacterial diversity study in the rumen of indigenous highland Zhongdian yellow cattle showed that Shannon indices were higher than those of rumen of lowland Jiangcheng yellow cattle. This work also reported a higher abundance of Firmicutes and Bacteroidetes, as well as enrichment for *Prevotella*, *Butyrivibrio*, and *Clostridium* in highland cattle rumen compared with that in low altitude cattle rumen, which was consistent with findings in other highland ruminants (Wu et al., 2020).

## Overview of introduced ruminants on the Qinghai–Tibetan plateau

Ruminants play major roles in economics and culture worldwide through production of dairy, meat, leather, and in labor (Eisler et al., 2014; Taye et al., 2017). In order to fulfill requirements for life on the plateau, non-native sheep and cattle breeds have been introduced and crossbred with indigenous breeds, then naturalized to the QTP. For example, Gansu alpine fine wool sheep (a cross between Tibetan and Xinjiang fine wool sheep), Small-tail Han sheep, and Suffolk sheep are all found in alpine pastures of the QTP, as are Jersey cattle, Sanhe cattle, Simmental cattle, and Holstein cows, in addition to some crossbred cattle used for meat and dairy production (Huang et al., 2016; Zhang et al., 2022).

In previous studies, we found that Jersey cattle showed adaptations to high altitude areas at both the miRNA and proteome levels (Kong et al., 2019), illustrating adaptive mechanisms through up-regulation of amino acid metabolism and sphingolipid metabolism (Kong et al., 2021). Zhang et al. (2022) found that rumen fermentation and bacteria characteristic of highland Sanhe heifers had a lower acetate-to-propionate ratio as alternative hydrogen electron acceptors for CH_4_ mitigation, a lower relative abundance of Actinobacteria, and higher relative abundance of Spirochaetae than that in rumen of lowland heifers. Cumulatively, these studies indicated that cattle introduced to the plateau also showed adaptations in phenotype and rumen fermentation microbiota. Other work by Sha et al. (2020) in a typical crossbred cattle-yak hybrid showed that Firmicutes were predominant in rumen, while several lignocellulose-degrading bacteria, such as *F. succinogenes* were also enriched.

During exposure to high altitudes, dairy cows reportedly exhibit deceased body weight and metabolizable energy used for milk production, together resulting in lower daily milk yields than those at low altitudes (Qiao et al., 2013; Saha et al., 2019). In addition to this production deficit, the high altitude and low atmospheric oxygen in this region can lead to development of brisket disease (BD) in Holstein heifers with mortality rates reaching almost 25% (Holt and Callan, 2007; Malherbe et al., 2012). Sanada et al. (2020) identified some gut microbes that could suppress the development of pulmonary arterial hypertension in a rat model of hypoxia. Further analysis identified potential microbial and metabolic markers in the rumen of Holstein cows suffering from BD, along with significantly decreased VFAs and rumen microbial diversity, concurrent with decreased abundance of *Ruminococcus* and *Treponema* (Gaowa et al., 2020; Yao et al., 2021). Unlike the long-term adaptations developed by indigenous ruminants, these introduced ruminants are still stressed by the high plateau environment. In future applications of these overall findings, differences in genetic background among stressed and non-stressed together with indicator rumen microbes, could be used to identify biomarkers for breed selection and production in crossbred livestock for the QTP. However, in contrast with shifts in microbiota detected after the onset of significant BD symptoms, microbial markers that appear when oxygen saturation of blood initially drops, prior to severe symptoms, may be more informative for accurate diagnosis and preventive interventions, which will be explored in future studies of this issue. In addition, since microbial colonization of ruminants early in life greatly impacts later animal productivity and health (Liu et al., 2017; Dill-McFarland et al., 2019), it is necessary to establish a clear picture of the phylogenetic composition, interactions, and maturation of rumen microbiota as a whole in dairy calves on the QTP. This enhanced understanding will help improve the health and productivity of ruminant livestock that are poorly adapted to life on the plateau.

Introduced cattle and sheep have been used as experimental controls in comparisons of rumen microbiota between indigenous and introduced ruminants raised in close proximity in the QTP. Huang et al. (2016, 2021) have demonstrated that rumen archaea and bacteria differ among indigenous and introduced ruminants (yak and Tibetan sheep vs. cattle and crossbred sheep). Notably, methylotrophic Methanomassiliicoccaceae are the predominant archaeal group in all tested ruminants in the plateau, whereas *Methanobrevibacter* is typically found in greater abundance in ruminants, globally. In bacterial analysis, two significant enterotypes affiliated with uncultured Ruminococcaceae and *Prevotella* were dominant in the indigenous and introduced ruminants, respectively. A screen of fecal microbiota in yak, cattle, yak-cattle hybrids, and Tibetan sheep from different eco-regions of the QTP by Wang X. et al. (2022) identified two enterotypes based on the prevalence of Ruminococcaceae UCG-005 or *Acinetobacter* which were closely related to diet and environment.

Protozoa are also present in the rumen of most domesticated ruminants, and play key roles in the digestion and fermentation of feed components. These protozoa are also significantly correlated with methanogens that contribute up to 37% of CH_4_ emissions from rumen (Finlay et al., 1994). Huang and Li (2018) examined rumen methanogens and protozoan communities in Tibetan sheep and Gansu alpine fine wool sheep grazing on the QTP and reported higher levels of *Methanobrevibacter millerae* than the more commonly detected *M. gottschalkii* in lowland ruminants. Additionally, holotricha protozoans comprised a lower proportion (1.1%) of total rumen protozoans of Tibetan sheep than introduced sheep, *Entodinium* (70.0%) serving as the predominant genus in Tibetan sheep and *Enoploplastron* (48.8%) dominating in Gansu fine wool sheep. A comparison of rumen microbiota between Tibetan sheep with three different introduced sheep reported by Chang et al. (2020) indicated that Ruminococcaceae and *WCHB1–25* were the most abundant families in the introduced sheep group, whereas Spirochaetaceae, S24–7, Prevotellaceae, Barnesiellaceae, and Succinivibrionaceae were the most abundant families in Tibetan sheep, further suggesting that fluctuations in rumen bacteria can be driven by variations in the host. However, a lack of studies investigating shifts in rumen microbiota sheep introduced at different altitudes prevents further conclusions regarding the effects of altitude on rumen microbiota in these animals.

The QTP is characterized by low temperatures and hypoxia, and hypoxia-inducible factors are likely to play an important role in the adaptation to high altitude hypoxia by Tibetan sheep, yaks, and introduced sheep (Qiu et al., 2012; Wang Y. et al., 2017; Wang F. et al., 2022; Zhao et al., 2021). Pral et al. (2021) recently showed that a hypoxic environment in the intestine can induce the expression of several hypoxia-inducible factor 1 (HIF-1) target genes in intestinal epithelial cells, consequently impacting their metabolism, barrier function, and survival. Specific gut microbes in rats have been correlated with elevated butyric acid levels in SCFA fatty acid profiles. This finding suggests the possibility that microbiota may activate hypoxia/HIF-1 during adaptation to the high altitudes in rats. Future work will examine if this potential cross-talk-mediated activation of hypoxia occurs in the host rumen microbiota in ruminants on the QTP. The development of host genetics and microbiome associations studies (Weissbrod et al., 2018) has led to improvements in microbial genome-wide association analysis (mGWAS) to overcome vagaries stemming from weak associations between microbiome host genotype. These improvements may help to understand shared microbiota-host adaptations to the plateau.

## Conclusion

Overall, there is a rapidly accumulating body of evidence in high altitude rumen microbiota studies that support three predominant driving factors in adaptation of indigenous ruminants to the QTP: increased VFA production, enhanced fiber degradation, and lower CH_4_ production. These three factors are indicators of higher efficiency energy harvest and nutrient utilization by microbiota that can sustain the host during nutrient deficit. While these findings support the importance of host-rumen microbe interactions, numerous questions persist regarding the mechanisms by which rumen microbiota facilitate host nutrient acquisition under the multiple stress conditions endemic to the QTP. Here, we systemically reviewed the dynamics of rumen microbiota in both indigenous and introduced ruminants (including gut microbiota of wild ruminants). These cumulative studies show that microbiota structure and functional enrichment shift in response to seasonal environmental cues, especially nutritionally poor forage materials and cold, to potentially enhance nutrient harvesting and increase the efficiency of host energy utilization. Future progress toward understanding ruminant adaptation to high altitudes will require the integration of phenotypic data with multi-omics analyses (e.g., mGWAS) to identify host-microbiota co-evolutionary adaptations enabling survival on the QTP.

## Author contributions

BL, ZL, and YW conceived the study. BL and GJ drafted the manuscript. BL, ZL, GJ, XXZ, JZ, QX, XLZ, NJ, and DW coordinated in data collection and created figures. All authors contributed to the critical revision of the manuscript, read, and approved the final manuscript.
